# Simultaneous surgical treatment tactics of acute destructive cholecystitis combined with choledocholithiasis: A case report

**DOI:** 10.1016/j.ijscr.2020.04.081

**Published:** 2020-05-12

**Authors:** Dauren T. Zhumatayev, Аbilay N. Baimakhanov, Mazhit K. Abdykadyrov, Dauren A. Nurmakov, Aidar D. Raimkhanov, Аlibek M. Smagulov, Nurken M. Abdiyev

**Affiliations:** a Department of Surgery, Asfendiyarov Kazakh National Medical University, 050012 Almaty, Kazakhstan; b City Clinical Hospital No. 4, 050054 Almaty, Kazakhstan

**Keywords:** Cholelithiasis, Cholecystitis, Endoscopic papillosphincterotomy, Laparoscopic cholecystectomy, Case report

## Abstract

**Introduction:**

The widespread development of minimally invasive treatment methods have expanded the choices and options available to surgeons to manage patients suffering from cholelithiasis and its complications, including choledocholithiasis. As new surgical strategies are developed and become widely available, there is an ongoing debate as to which surgical strategies are optimal for the management of acute cholecystitis and concomitant choledocholithiasis. The treatment of patients in this category should be carried out according to clear criteria, taking into consideration the patient's condition, concomitant diseases, the size of calculi in the common bile duct as well as the resources of the medical institution.

**Case presentation:**

We present the clinical case of a 65-year-old female with cholelithiasis, acute gangrenous cholecystitis, choledocholithiasis, and obstructive jaundice. She was treated with endoscopic retrograde cholangiopancreatography (ERCP) with endoscopic papillosphincterotomy (EPST) and laparoscopic cholecystectomy (LCE) in a one-stage operation.

**Discussion:**

Several sources recommended performing LCE after ERCP with EPST in acute cholecystitis when complicated by choledocholithiasis to reduce the conversion rate and the risk of recholedocholithiasis.

**Conclusion:**

Simultaneous resolution of acute destructive cholecystitis complicated by choledocholithiasis in a one-stage operation is accompanied by a decrease in emotional and psychological trauma, also leads to early rehabilitation of patients and, thereby, reduces overall postoperative disability and morbidity.

## Highlights

• Acute destructive cholecystitis complicated by choledocholithiasis in elderly patients.
• A one-stage operation in acute cholecystitis when complicated by choledocholithiasis.
• Performance of LCE after ERCP with EPST in acute cholecystitis when complicated by choledocholithiasis.

## 1 Introduction

Choledocholithiasis is diagnosed in 10–15% of patients with acute calculous cholecystitis, and in 8–15% of patients under the age of 60, and in 15–60% in patients over 60 [1]. The surgical treatment of patients with acute cholecystitis complicated by choledocholithiasis is a laborious task in biliary tract surgery.

Complicated forms of cholelithiasis (choledocholithiasis, cholangitis, obstructive jaundice) and its various forms of progression, as well as the prevalence of elderly patients involve the methods of diagnostics and choice of surgical treatment.

The choice of surgical tactics is still debated by surgeons (e.g. whether to perform the operations in a one-stage or two-stage operation, and whether to sanitise the biliary tract before, during or after cholecystectomy). The optimal surgical tactics for treating patients with cholecystocholedocholithiasis currently are not defined, and so treatment often depends on the characteristics of an individual clinic, its resources and experience of surgeons, endoscopists and radiologists [2]. The choice of treatment tactics remains relevant due to the advent of a large number of minimally invasive technologies, the possibilities of which are not fully disclosed [3] when applied to patients with this pathology.

In contemporaneous practice, the number of open surgical interventions on bile ducts has been reduced compared to the number of minimally invasive interventions, which is due to the development of laparoscopic, endoscopic, and transcutaneous treatment methods [4].

Nowadays surgeons have new opportunities in terms of choosing the type of surgical treatment for cholecystocholedocholithiasis due to the introduction of new approaches and the development of technologies in surgery.

In considering the best surgical approach for cholecystocholangiolithiasis, the choice is contingent on a number of general and specific factors, such as the degree of operational risk, the presence of complications of cholecystocholangiolithiasis, the form of cholecystitis, stone features (diameter, number), and concomitant diseases of the biliary papilla.

These factors determine the scope and the duration of the operation, as well as the number of treatment stages [5].

In this paper, we report on the successful application of simultaneous surgical treatment tactics in a one-stage operation for acute gangrenous cholecystitis complicated by choledocholithiasis, through the use of endoscopic retrograde cholangiopancreatigraphy, papillosphincterotomy (ERCP with EPST), followed by laparoscopic cholecystectomy (LC). The work has been reported in line with the SCARE criteria. This case was reported in accordance with the SCARE Guidelines [6].

## 2 Case presentation

The patient (65-year-old) was admitted expediently to the surgical department of the City Clinical Hospital No. 4 in Almaty with the complaints of severe pain in the right hypochondrium and epigastrium, nausea, bilious vomit, dry mouth, general weakness, and scleral icterus. Physical examination revealed bloating, tenderness on superficial and deep palpation in the right hypochondrium, with positive symptoms of Murphy, Ortner-Grekov, and Kerte.

At the time of hospitalization, a blood test showed high values of white blood cells – 18.4 × 10^9^/L, total bilirubin – 47.17 mg/dL, conjugated bilirubin – 20.19 mg/dL; there were signs of hepatorenal syndrome with an increased values of blood creatinine – 118.6 mg/dL and urea – 10.45 mg/dL (Table 1).

**Table 1:** Pre- and post-operative parameters.

	Day 1 Hospitalization	Day 4 Preoperative	Day 5 Postoperative	Day 8 Discharge
White Blood Cells (WBC), 10^9^/L	18.4	17.6	9.9	7.2
Alanine Aminotransferase (ALT), IU/L	22.12	59.26	35.29	20
Aspartate Aminotransferase (AST), IU/L	25.81	86.91	80.25	28.1
Total Bilirubin (TBIL), mg/dL	47.17	52.85	14.42	8.21
Conjugated Bilirubin, mg/dL	20.19	26.26	6.29	3.5
Blood Sugar, mg/dL	5.41	3.51	4.88	4.93
Blood Creatinine, mg/dL	118.6	109.8	68.3	66.9
Urea, mg/dL	10.45	10.21	4.61	2.24
Amylase, IU/L	49.6	40	45.5	43.3
Total Protein, g/dL	5.8	4.2	5.8	6.0

Ultrasound showed elongation of gallbladder with concomitant wall thickening and with many calculi in the gallbladder and choledochoectasia. A calculus in the terminal section of the common bile duct with a size of 0.8 × 0.7 cm was found. The preliminary diagnosis was that of cholelithiasis, acute cholecystitis, and choledocholithiasis, leading to an obstructive jaundice.

The patient categorically refused the proposed operation and signed a “refusal to consent” form. On the 4th day, the patient's condition worsened. As a result a panel of doctors was urgently gathered to discuss the patient's condition. There were signs of increasing intoxication as well as the obstructive jaundice. The patient was again offered the operation, and she was also aware that in case of refusal, complications of the disease can lead to death. The patient agreed to the surgery. After the preoperative preparation, on the 4th day, the patient was transferred to the operating unit. In the operating room the patient was under endotracheal anesthesia in the prone position. Endoscopic retrograde cholangiopancreatography with endoscopic papillosphincterotomy was performed (first stage). A shadow about 0.8 cm in size was revealed in the lower third choledochus. Common bile ductus (CBD) stone extraction was performed using a Dormia basket. A stone was extracted with dimensions of 1.0 × 0.8 cm. The flow of bile was restored after the extraction of the stone.

At the end of the procedure, the air was aspirated from the upper gastrointestinal tract (stomach, duodenum 12), and a nasogastroduodenal probe was installed under the control of the endoscope. The endoscope was removed. Then, the patient was in a supine position (second stage). A pneumoperitoneum was achieved after processing the surgical field using a Veress needle. An examination was made and an expansion of the initial sections of the small intestine was noted. The air was aspirated again from the intestine using the nasogastroduodenal probe. The trocars were installed at usual trocar placement. The upper part of the operating table was raised 20° and turned to the left to 15°. During the examination, the gallbladder was 12.0 × 6.5 cm, tense, swollen, and dark purple with dense perivesical infiltration. The cystic duct and cystic artery were isolated, clipped, and severed. A cholecystectomy from the neck subserous was performed, the gall bladder was removed, and a microirrigator was installed in the subhepatic space.

In the postoperative period, a comprehensive conservative treatment was performed. On the 7th day, the microirrigator was removed; ultrasound revealed a common bile duct length of 8 mm, and esophagogastroduodenoscopy showed transparent bile freely entering the duodenum through the large duodenal papilla. On the 8th day, the patient was discharged in satisfactory condition, with normal liver tests values (Table 1). At an outpatient follow-up appointment, 1 month after discharge, the patient had no complaints. She was satisfied with the treatment.

## 3 Results and discussion

**Fig. 1:**
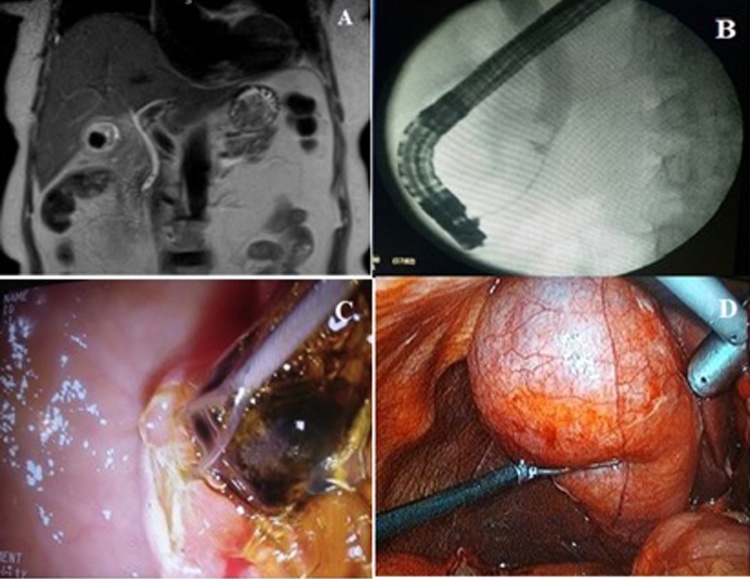
(A): MRCP revealed a CBD stone in the lower CBD and stones in the gallbladder. (B): Contrast examination of the CBD. (C): Intraoperative removal of the CBD stone by basket. (D): Intraoperative demonstration of gangrenous gall bladder.

The patient underwent the simultaneous treatment of acute destructive cholecystitis with concomitant choledocholithiasis well. There were no intraoperative complications. The duration of the endoscopic component – 30 min; the surgical component – 65 min. There was 10.00 mL of blood loss. After the operation, there was a noticeable decline in values of blood creatinine – 68.3 mg/dL, urea – 4.61 mg/dL, of white blood cells – 9.9 × 10^9^/L, total bilirubin – 14.42 mg/dL, and conjugated bilirubin – 6.29 mg/dL (Table 1). The patient was treated in hospital for 8 days (Fig. 1).

The first study, which reported the importance of the time interval between ERCP and LCE, was published in 2005 [7]. Several sources recommended conducting LCE immediately after ERCP with EPST in acute cholecystitis complicated by choledocholithiasis to reduce the risk of conversion and acute destructive cholecystitis [8,9]. If there is a time interval between the endoscopic and surgical stages of treatment, it leads to the conversion in 20% of patients undergoing ERCP with EPST [10]. Most studies have reported that LCE in the early stages after ERCP is the most optimal, cost-effective method, and reduces the rate of conversion. Technical difficulties have been noted with delayed LCE, principally due to fibrotic changes, adhesions and scar changes in the gallbladder that occur over time [11]. Salman et al. [12] suggested performing early LCE after ERCP with EPST, as delayed LC is associated with higher conversion rate and therein an increase in duration of hospital stay, in tandem with an increased rate of transitions to open surgery and postoperative complications. Some authors believe that for patients with acute cholecystitis and concomitant choledocholithiasis, early LCE is desirable due to the high risk of complications associated with acute inflammation of the gallbladder [13,14]. Wild et al. [15] compared the surgical tactics of performing cholecystectomy and ERCP separately, with the surgical tactics of performing cholecystectomy and ERCP on the same day, and demonstrated increased safety, efficacy, and cost-benefit ratio for the latter. Friis et al. [16] in their systematic review showed that early laparoscopic cholecystectomy does not increase mortality, the incidence of perioperative complications, or the length of stay, and on the contrary, it reduces the risk of reoccurrence and progression of the disease. As a result, they concluded that, ideally, for this category of patients, it is preferable to perform cholecystectomy within 24 h after ERCP, or at least in the first few days. Nonetheless, some authors have described a “wait and see” policy after resolving choledocholithiasis in patients who do not have an acute inflammatory change in the gallbladder [17]. Possible complications in the waiting period after an ERCP with EPST are acute destructive cholecystitis, recholedocholithiasis, cholangitis, biliary pancreatitis, and recurrent biliary colic. They lead to a decrease in the quality of life and working capacity.

The technical results of the proposed surgical tactics are: successful resolution of acute destructive cholecystitis complicated by choledocholithiasis by simultaneous surgery; reduction of the invasiveness of the operation; reduction of moral and psychological burden; a low frequency of intra and postoperative complications; use of smaller doses of drugs; the possibility of early rehabilitation of patients in the postoperative period; a significant reduction of the hospitalization period; reduction of postoperative disability time.

## 4 Conclusion

We believe that this approach should be applied only to a certain category of patients; that is for those who were admitted with acute destructive cholecystitis complicated by choledocholithiasis and need an emergency resolution of both complications of cholelithiasis.

## Declaration of Competing Interest

We confirm that the manuscript has been read and approved by all named authors and that there are no other persons who satisfied the criteria for authorship but are not listed. We further confirm that the order of authors listed in the manuscript has been approved by all of us.

## Sources of funding

The authors received no specific funding for this work.

## Ethical approval

Ethical Approval was given by Ethnical Committee of Non-Commercial Joint Stock company «S.D. Asfendiyarov Kazakh National Medical University», Protocol No. 8(87) as of 13th September, 2019.

## Consent

Written informed consent was obtained from the patient for publication of this case report and accompanying images. A copy of the written consent available for review by the Editor-in-Chief of this journal on request.

## Author contribution

Zhumatayev Dauren Talgatuly – Performed the operation. Participated in writing the case report.

Baimakhanov Abylai Nyiatovich – Performed the operation. Candidate Doctor of Medical Science, Professor of Chair of Surgery. Participated in data collection, data analysis, writing the case report.

Abdykadyrov Mazhit Kulikovich – Participated in data collection, data analysis, writing the case report.

Nurmakov Dauren Amanovich – Participated in data collection, data analysis, writing the case report.

Raimkhanov Aidar Duysenovich – Participated in data collection, data analysis, writing the case report.

Smagulov Alibek Mukhamedzhanovich – Participated in data collection, data analysis, writing the case report.

Abdiyev Nurken Mahamashevich – Participated in data collection, data analysis, writing the case report.

## Registration of research studies

This case report is not first-in-man study.

## Guarantor

Dauren T. Zhumatayev.

## Provenance and peer review

Not commissioned, externally peer-reviewed.
